# Sideroblastic changes of the bone marrow can be predicted by the erythrogram of peripheral blood

**DOI:** 10.1111/j.1751-553X.2009.01185.x

**Published:** 2010-06

**Authors:** A ROVÓ, G STÜSSI, S MEYER-MONARD, G FAVRE, D TSAKIRIS, D HEIM, J HALTER, C ARBER, J PASSWEG, A GRATWOHL, A TICHELLI

**Affiliations:** Hematology, University Hospital of BaselBasel, Switzerland

**Keywords:** Ring sideroblasts, sideroblastic anemia, myeloid neoplasms, myelodysplastic syndromes, scattergram, erythrogram

## Abstract

The diagnosis of sideroblastic anemia is based on bone marrow aspiration, and the detection of ring sideroblasts (RS) in iron staining. The finding of laboratory parameters to approach this diagnosis still remains a great challenge. In this study, we analyzed the value of a specific erythrogram pattern from peripheral blood, produced by the ADVIA®120 cell counter, to predict sideroblastic changes in the bone marrow. In a two step-design study, we first showed that 32/38 consecutive patients reporting ≥15% RS had such a pattern in the erythrogram. In the second step, we prospectively identified over a period of 32 months 21 patients with this typical erythrogram; 20/21 had ≥15% RS in the bone marrow. Hence, by this validation, we confirm that the erythrogram is highly predictive of RS in the bone marrow. The interpretation of the erythrogram should become daily practice in hematology to improve the efficacy to detect sideroblastic changes.

## INTRODUCTION

The presence of ring sideroblasts (RS) in the bone marrow aspiration is the hallmark for hereditary and acquired sideroblastic anemia. Sideroblasts are visible with Perl’s Prussian blue staining under microscopic examination of bone marrow. In adult patients, RS are highly suggestive of a myelodysplastic syndrome (MDS) or another type of myeloid neoplasm with RS, when other causes have been ruled out. In acquired sideroblastic disorders, anemia is usually normo- or macrocytic with a variable population of hypochromic red blood cells (RBCs) on the blood smear, and the occasional presence of circulating erythroblasts. However, none of these changes in peripheral blood is distinctive enough to predict the diagnosis of sideroblastic anemia.

New generation hematology systems supply essential information on blood cells, complementing microscopic cell examination. The RBC scatter plots, generated by the automated ADVIA®120 hematology analyzers (Bayer Diagnostics, Tarrytown, NY, USA), provide a visual representation of RBC characteristics, based on the volume and hemoglobin concentration. These erythrograms show a characteristic pattern in diverse conditions such as iron deficiency, thalassemia, autoimmune hemolytic anemia and spherocytosis, and their use is valuable in daily practice (Ricerca *et al.*, 1987; Pati, Patton & Harris, 1989; Macdougall *et al.*, 1992; d’Onofrio *et al.*, 1992; Robertson *et al.*, 1992; McKay *et al.*, 1993; Kutter *et al.*, 2002).

**Figure 1:**
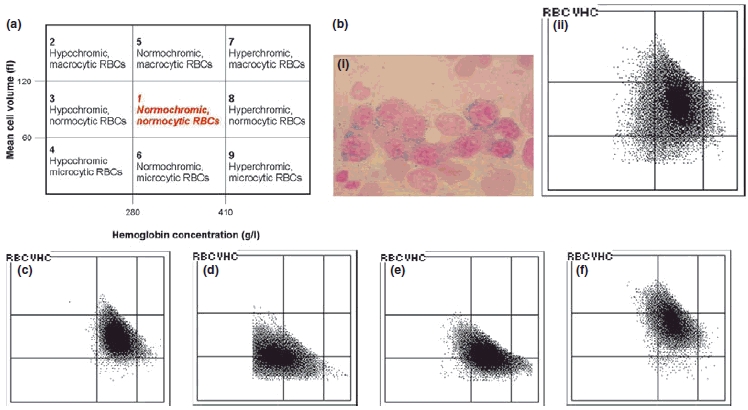
The erythrogram is a graphical representation of red blood cells (RBCs) produced by the automated cell analyzer ADVIA®120 (Bayer), based on cell by cell measurements of hemoglobin concentration (abscissa), and red cell volume (ordinate). (a) Diagram of an erythrogram divided into nine areas, according to the red cell volume and hemoglobin concentration. Normally the erythrocytes are located within the area 1 (normochromic, normocytic RBC). RBCs located on the left side of the first vertical line passing at 280 g/l are hypochromic. RBCs located on the right side of the second vertical line passing at 410 g/l are hyperchromic. RBCs located above the first horizontal line passing at 120 fL are macrocytic; RBCs located below the second horizontal line, assigned at 60 fL are microcytic. The erythrogram offers a sensitive and specific method for detection of minority of RBC populations, and provides a typical pattern which assists in differentiating between diseases such as iron deficiency anemia, thalassemia minor, and macrocytosis. (b) Patient with sideroblastic anemia: (bi) Iron staining of the bone marrow cytology with RS. (bii) Erythrogram of peripheral blood from the same patient showing a typical RS-pattern, with the RBCs largely scattered over eight of nine areas (all except area seven). In comparison erythrograms with different pattern: (c) normal erythrogram, (d) iron deficiency anemia; the RBCs are mainly moved to area four, with microcytic, hypochromic RBCs, (e) thalassemia minor; the RBCs are moved mainly to area six with microcytic, normochromic RBCs, and (f) macrocytosis; the RBCs are mainly moved to area five, with macrocytic, normochromic RBCs.

We observed a characteristic erythrogram in patients with sideroblastic anemia, consisting of an unusual broad scatter distribution of the red cells in respect of their volume and hemoglobin concentration, with a substantial number of dots overlapping onto the hypochromic and the microcytic quadrants. This erythrogram is clearly distinctive from iron deficiency, megaloblastic anemia or MDS without RS (Figure 1). We sought to confirm the diagnostic value of the erythrogram to predict sideroblastic changes in the bone marrow.

## DESIGN AND METHODS

This was a single center study with a two step model design. In the first step, we reviewed all reports of bone marrow cytology performed in the Hematology Laboratory of the University Hospital of Basel between January 2004 and June 2006. Any case with ≥15% RS in the iron staining (MDS, other myeloid neoplasms, or reactive forms of sideroblastic anemia) and having a peripheral blood count performed with an ADVIA®120 system, were included in the study. These cases were compared with a control group of 30 patients with a myeloid neoplasm presenting dysplastic features without RS. We defined as RS-pattern (Figure 1bii) an erythrogram with a broad distribution of the RBCs over nearly eight of the nine areas of the scattergram (Figure 1a), with dots spread over the macrocytic, microcytic and hypochromic areas. All other erythrogram patterns were considered as non-RS-pattern. The erythrogram as well as numerical RBC parameters were compared between both groups.

The second step of the study aimed to validate the RS-pattern. Therefore, from January 2006 to June 2008, we collected prospectively all cases with a RS-pattern performed in the Hematology Laboratory and evaluated their predictive value for sideroblastic changes in the iron staining of bone marrow cytology. The Hematology Laboratory operates as a central Lab for the whole University Hospital, performing a mean of 600 automated peripheral blood counts daily. For this second step, the technicians were trained to identify the RS-pattern during their routine work. From each newly detected peripheral blood count with a RS-pattern, a smear of peripheral blood was performed and together with the erythrogram forwarded to the physician in charge of the laboratory to initialize the required investigations. All patients with representative cytology smears of the bone marrow were included for this analysis in the validation group. Patients with repetitive evaluations were considered only once. This study has the approval of our Institutional Review Board

A Mann–Whitney *U*-test was used to show numerical differences between groups. In all statistical procedures, *P*<0.05 was considered the level of significance. Statistical analysis was performed using SPSS statistical software (spss for Windows, Release 14.0; SPSS, Inc., Chicago, IL, USA).

## RESULTS AND DISCUSSION

In the first step of the study, 38 cases with newfound RS in the bone marrow and available ADVIA peripheral blood analysis were identified within 3285 evaluated bone marrow results. Thirty two (84%) of the 38 cases had the typical RS-pattern in the erythrogram. In the control group none of the 30 patients without RS (*P* < 0.0001) had such a pattern. This typical RS-pattern has a numerical background (Table 1). There were significant differences in RBC-indices between patients with and those without RS. Only few RS-patients showed microcytic (6/38), or hypochromic anemia (7/38) or had a decreased content of hemoglobin of the reticulocytes (CHr) (2/38). These findings contrast with the high number of RS-patients with an increased percentage of hypochromic RBC (hypoRBC). RS-patients showed consistently ≥3% hypoRBC (26/38, 68%) compared to patients without RS (7/30; 23%). The combination of increased hypoRBC and low CHr is usually closely related to iron deficiency (Fishbane *et al*., 1997, 2001; Tessitore *et al.*, 2001; Kotisaari *et al.*, 2002; Radtke *et al.*, 2005). In our cohort with RS, we have the particular constellation of increased hypoRBC, but normal CHr (25/26 RS-patients). An increased percentage of hypochromic erythrocyte has been previously described in patients with refractory anemia and RS (Ljung, Back & Hellstrom-Lindberg, 2004; Murphy *et al.*, 2006). Another constant feature in patients with RS-pattern was the increased red cell distribution width (RDW) with values ≥19% in 22/38 (58%). In contrast, a RDW ≥19% was observed only in 3/30 (10%) patients without RS. Despite significant differences found between patients with and without RS, none of these numerical parameters used without the erythrogram was useful in the daily practice to predict sideroblastic changes in the bone marrow. Indeed, first, for each individual parameter, there was a substantial overlap between patients with and without RS. Secondly, we looked for a useful tool in the daily practice allowing to suspect the presence of RS at first sight. None of the numerical parameters fulfilled these criteria.

**Table 1:** Patient’s characteristics and RBCs values. In the first step analysis, comparison of patients with and without RS; in the second step analysis, validation with patients presenting a typical RS-pattern

	Analysis of the first step			Second validation step	
	With RS	Without RS	*P*-value (with RS *vs.* without RS)	Validation	*P*-value (with RS *vs.* validation group)
*n*	38	30		21	
Female/male	18/20	9/21		9/12	
Median age (range)	67.5 (23–95)	64 (23–91)		63 (42–85)	
Diagnose					
AML	6	6		4	
NOS	3	–		3	
t-myeloid neoplasm	–	–		1	
AML with MLD	3	6		–	
MDS	21	21		11	
RA	–	3		–	
RARS	6	–		8	
RCMD	9	3		–	
RAEB-1 and RAEB-2	6	12		3	
del (5q):	–	1		–	
Unclassifiable	–	2		–	
MPN	3	–		4	
CML-AP	2	–		2	
PMF	1	–		1	
PV	–	–		1	
MDS/MPN	6	3		1	
RARS-T	4	–		–	
CMML:	2	1		–	
Unclassifiable	–	2		1	
Others	2	–		1	
Hemoglobin, g/l	95 (61–151)	95 (68–144)	0.72	95.5 (80–121)	0.98
MCV, fL	91.2 (75.4–113.7)	99.7 (84–116.7)	0.001	93.6 (78.1–100.7)	0.53
MCH, pg	30.8 (22.8–37)	34.7 (27.6–39.9)	0.0002	31.5 (26.2–36.3)	0.64
MCHC, g/dl	335.5 (265–365)	340.5 (307–367)	0.203	336 (300–346)	0.72
RDW, %	21.4 (14.6–33.8)	17.1 (13.8–28.6)	0.0002	22.6 (19–28)	0.019
hypoRBC,%	5.3 (0.3–78)	1.3 (0.1–21.2)	0.00006	6.7 (3.3–20.9)	0.25
Retic (×10^9^/l)	73.6 (9–251)	39.4 (0.5–164.9)	0.002	49.9 (5.4–209.6)	0.14
CHr, pg	33 (25–51)	37 (28–41)	0.002	33.5 (27–38)	0.67
Erythrogram pattern					
RS-pattern	32	0	<0.000001	21	
Any other pattern	6	30		0	
≥15% of RS in BM	38	0		20	

RS, ringed sideroblasts; AML, acute myeloid leukemia; NOS, not otherwise specified; t-myeloid neoplasm, therapy-related myeloid neoplasm; AML with MLD, AML with multilineage dysplasia; MDS, myelodysplastic syndromes; RA, refractory anemia; RARS, refractory anemia with ring sideroblasts; RCMD, refractory cytopenia with multilineage dysplasia; RAEB-1 and RAEB-2, refractory anemia with excess blasts 1 and 2; del (5q), associated with isolated del (5q); MPN, myeloproliferative neoplasms; CML-AP, CML BCR-ABL1 positive in accelerated phase; PMF, primary myelofibrosis; PV, polycythemia vera; RARS-T, RARS associated with marked thrombocytosis; CMML, chronic myelomonocytic leukemia; MCV, mean corpuscular volume; MCH, mean corpuscular hemoglobin; MCHC, mean corpuscular hemoglobin concentration; RDW, red cell distribution width; hypoRBC, hypochromic erythrocyte; Retic, absolute reticulocytes; CHr, content of hemoglobin of the reticulocytes; BM, bone marrow cytology.

Six RS-patients did not have an erythrogram with a RS-pattern (2 Acute myeloid leukemia, 2 MDS/myeloproliferative neoplasms, 1 MDS, 1 hemophagocytosis). All six patients had increased iron stores, 5/6 were anemic and did not have the typical numerical parameters (4/6 high MCV; 5/6 < 20% RDW; 5/6 < 1.1% hypoRBC).

**Figure 2:**
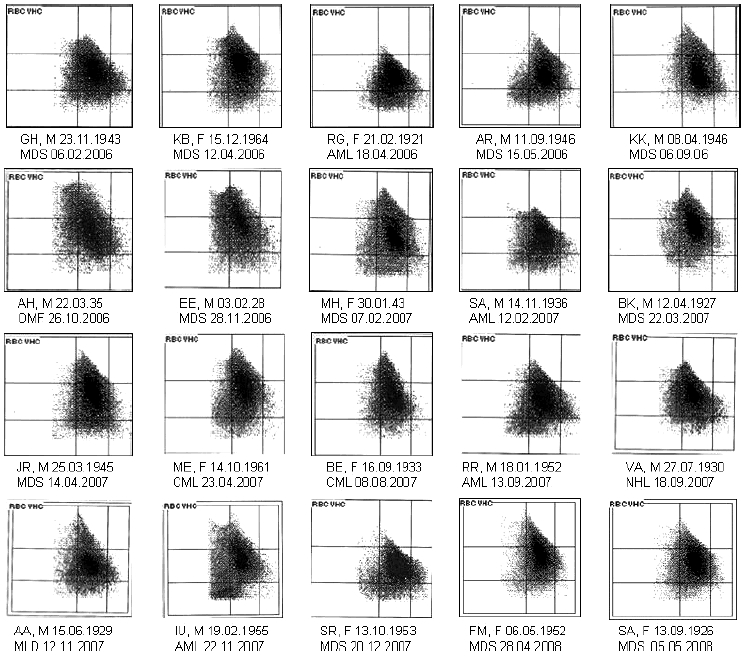
Erythrograms of all 20 patients of the validation group with a typical RS-pattern and ringed sideroblasts in the bone marrow.

In the second step of the study, we compared the validation group with the initial RS-patients. We identified prospectively 28 cases with a RS-pattern; 7/28 cases were excluded because they had a dry tap in the bone marrow aspiration. We found RS in the bone marrow of 20/21 evaluable cases, with a median of 50% RS (range 15–75%) Figure 2. All of them had increased iron stores. The patient with a typical RS-pattern, but without RS in the bone marrow, had depleted iron stores. As expected, RBC-indices of both groups were similar (*P* > 0.10) (Table 1). Hence, by this validation, we confirm that the peripheral blood erythrogram is highly predictive of RS in the bone marrow.

Siderotic granulations within the mitochondria form the hallmark of RS. Aspirate smears represent the gold standard for assessing RS. Mitochondrial iron is dissolved during treatment with chelation and decalcification agents. Therefore, RS remain unstained in paraffin sections obtained by decalcified tissue. They can only be identified in undecalcified material of plastic sections or with the use of special staining techniques (Tham & Cousar, 1993). Thus, in patients with fibrosis and dry tap in marrow aspiration, sideroblastic changes can be easily missed when no special attention is brought. According to our results, even in patients with dry tap, we can assume the presence of sideroblastic changes when patients have a typical RS-pattern. Moreover, if erythroblasts are present in peripheral blood, an iron staining could support this assumption by detecting circulating RS.

We demonstrated here the value of the erythrogram as a simple diagnostic tool in the routine laboratory analysis. It works as a ‘fingerprint’ and can evoke a diagnosis at ‘first look’ and consequently drive the investigations to confirm our suspicion. Despite that, a size distribution histogram allows identifying dimorphic red blood cell populations, a two dimensional erythrogram has a great specificity for suspecting sideroblastic changes. As sideroblastic anemia is frequently a normocytic, normochromic anemia, it can be easily misinterpreted with anemia of chronic disorder. Nine of 21 patients from the validation group presented normocytic and normochromic anemia without neutropenia and thrombocytopenia. In these patients, the erythrogram (RS-pattern) was a strong argument to think about an underlying myeloid neoplasms and to perform a bone marrow investigation. As for iron deficiency, thalassemia or macrocytosis, the erythrogram pattern has also a great visual impact to detect sideroblastic changes. Additionally, the RS-pattern is supported by the combination of the following RBC-indices: normal MCV and MCHC, RDW ≥19%, hypoRBC ≥3% and normal CHr.

In conclusion, this is the first study evaluating systematically the value of the erythrogram of peripheral blood as a diagnostic tool in daily practice to predict sideroblastic changes in the bone marrow. These results confirm the great value of the erythrogram as part of the routine evaluation of peripheral blood count.
